# Study on Effect of Leather Rigidity and Thickness on Drapability of Sheep Garment Leather

**DOI:** 10.3390/ma14164553

**Published:** 2021-08-13

**Authors:** Hafeezullah Memon, Eldana Bizuneh Chaklie, Hanur Meku Yesuf, Chengyan Zhu

**Affiliations:** 1 College of Textile Science and Engineering, International Institute of Silk, Zhejiang Sci-Tech University, Hangzhou 310018, China; 2 Ethiopian Institute of Textile and Fashion Technology, Bair Dar University, Bahir Dar 6000, Ethiopia; elduyod@gmail.com (E.B.C.); 419005@mail.dhu.edu.cn (H.M.Y.); 3 College of Textiles, Donghua University, Shanghai 201620, China

**Keywords:** leather, drape coefficient, leather rigidity, drapability, leather thickness

## Abstract

Understanding the performance and behavior of garment leathers provides valuable inputs for the design and production of leather garments. The drape is one of the important properties associated with garment fitness quality and appeal. This study aims to show how the independent variables flexural rigidity and thickness affect the dependent variable drapability. Nowadays, studies on the drape of garment leathers are scarce. In this work, the drape coefficient (DC) was measured for sheep garment leather, which influences the garment drapability, such as flexural rigidity in the range of 9.2 to 22 and thickness in the range of 0.64 to 0.96. The average DC was calculated in the range of 47.35 to 69.9% for the selected sheep leathers from four samples. The drapability of the garment leather was determined using the DC. Flexural rigidity and thickness have been shown to have a considerable influence on the DC, while they do bear a significant relationship to the DC. The results of this study can be used as an elementary tool for leather selection of appropriate materials for garments.

## 1. Introduction

Leathers for garments can be manufactured from almost all types of raw stock [1]. Nevertheless, the widely used raw material is sheepskins for their histological characteristics [2]. All the other skins such as goat skins, cow skins, and calf skins are also widely used, and the thickness range varies from 0.6 to 1.0 mm [3]. The qualities of the garment leathers are always required to be similar for their specific purpose and end use [4]. Some of the quality parameters and comfort parameters and the methodologies to be followed in leather processing are discussed below.

There are different properties that garment leather should have, such as lightweight, fastness (to rub, light, wash, etc.), durability, drapability, etc. [5]. On the other hand, the drape is the most significant garment feature, since it contributes to wear comfort [6]. This feature refers to the leather’s capacity to fall in a similar manner to textile and conform to the body’s shape when worn [7]. It is an inherent and essential quality for any leather to be called garment leather [8]. Whenever leather is having a cloth-like fall, it is said to have the quality of drape. The leathers require being as flexible as cloth with the utmost degree of softness without any firmness. Significantly less filling and more fat-liquoring will increase the drape quality of the leather [9]. Leather drapability is a morphological property that occurs when leather is hung down for gravity reasons. It is one of the essential indicators when measuring close-fitting clothing [6]. Therefore, good drapability is necessary for garment leathers [10]. The drapability of leather is closely related to the clothing classification [11]. There are two ways to examine leather drapes: using your senses or using testers (commonly used) [12]. A circular sample with a specific area is placed on a sample clamping plate, with their centers overlapping, causing the drape sample to droop along with the circular plate due to gravity [13].

According to the research done to date [6], the leather drape is determined mainly by the leather thickness, and rigidity/stiffness properties, with leather tensile and weight also of some importance. Therefore, to be able to determine the leather drape requires that the precise relationship between leather drape and leather rigidity /stiffness and thickness is known and that their effects on the leather physical property are also known [14]. In addition, leather garment drapes will be considered from the drape coefficient (DC) perspective only [15]. The bending length of the leather can determine the rigidity of the leather. For a better knowledge of drapability, it is essential to look into and grasp the fundamental bending behavior of leather [15]. The DC determines drapability; if the DC becomes between 30 and 80, the leather could be used for apparel [16]. It is well known that there are different hierarchical levels of leather within leather attach, and this is especially true for leather that has not been staked or fat-liquored [17]. The rigidity and stiffness of leather can be related to its bending length, as reported by [15]. The other main thing that must be noticed in the selection of leather is the effect of leather tanning material on the leather thickness and stiffness property [18]. Vegetable tanning material gives more thickness, fullness, roundness, etc. [19], whereas chrome gives empty, flexible, and soft leather [20].

This research aimed to show the actual effects of thickness and stiffness variability on leather drape quality difference by taking different leathers to measure all variables and analyze the result. Herein, leathers with vegetable and chrome tanned or re-tanned to reach each factor level in one sample leather have been studied. This helps set the optimum levels of independent variables thickness and flexural rigidity and optimize dependent variable drapability.

## 2. Experimental

### 2.1. Materials

Four types of sheep leather for the garment were reached from the EiTEX leather laboratory store. Among them, Leather 1 is chrome tanned leather; Leather 2 is vegetable tanned chrome re-tanned; Leather 3 has lower thickness higher rigidity; this leather is chrome tanned, vegetable re-tanned type of leather, and Leather 4 is vegetable-tanned.

Table 1 shows standard and measured values of independent variables (thickness and rigidity) that influence the dependent variable drape coefficient of the garment. If measured values from specimens in between their minimum and maximum limit levels of Indian standard IS 6490, the sheep leather samples can be used for apparel applications. Therefore, the specimens used for this research can be used for apparel applications.

### 2.2. Method

Factorial designs are commonly employed in engineering experiments involving two factors where the combined effect of the factors on a response must be studied [21]. A factor’s effect is defined as the change in reaction caused by a change in the factor’s level [22]. The effects of leather thickness and rigidity on the separation force are next investigated using a factorial design [22]. A factorial design is commonly used to investigate the impacts of corresponding components, material qualities, and establish the best study circumstances, among other things. This includes ANOVA, regression analysis, model significance, model adequacy, and fit statistics using design expert software.

### 2.3. Research Design

This study follows an experimental research design (Full Factorial Design) because experimental research follows a scientific approach, where it includes a hypothesis, a variable that the researcher can manipulate, and the variable that can be evaluated, calculated, and compared. The study aims to distinguish the optimal effect of leather rigidity and thickness on garment drape property to select the appropriate leather property, which gives better results to recognizers obtained through experimental studies. These also required extensive experimentation, which involves examining the leather’s physical properties through laboratory experimentation and giving better results, showing the effect of factors on leather drapes.

This testing works together with laboratory assistants. The conclusion of the cause-and-effect research reveals that two elements directly impact the leather’s drapability. They are flexural rigidity and thickness of the material. Flexural rigidity is a measure of the stiffness of leather and is related to the bending length of the specimen due to gravitational force. Thickness is the distance through the leather, as distinct from width or height [23]. In a factorial experiment, these variables are assigned as independent variables.

Technical experience and understanding of operating and various experiments are required to set the factors in this experiment. It is known from their experiences that if all parameters are set too low, the drapability increases [24]. However, if these parameters are set too high, the drapability will be low because the DC will also be high. To avoid unfavorable outcomes in the experiment, proper samples of garment leather with the same thickness and rigidity are necessary. The expected ranges of factors are presented based on the experiment. Since all factors have two levels, a 22 factorial design is used. Each run is performed with three replicates at random, and the data are assessed at a significant level of 1 to achieve a very low type III error.

### 2.4. Thickness

The thickness of samples was measured with a digital thickness Gauge TF121C as leather has different thicknesses in different portions such as the shoulder (the thickest part), butt (the thicker area next to the shoulder area), belly (poor portion), and shank. Therefore, each leather sample was measured fifteen times to get a more accurate measurement, and finally, we used the average lower result of a thickness as a minimum and the average higher result as a maximum level.

Table 2 shows that the two samples (L1 and L3) have the same thickness and rigidity level, while the other two samples (L2 and L4) have the same thickness and rigidity levels. Due to these reasons, average measurements of two leather kinds, either L1 and L3 or L2 and L4, were used for minimum and maximum thickness levels.

### 2.5. Bending Length

Bending length is the ability of the leather that can be bent somewhere [25]. The Shirley stiffness tester, which comprises a platform with smooth low friction and flat surfaces, was used to measure the bending length of samples. Specimens are made from sheep leather. Three specimens were cut from four kinds of leather (2.5 × 21.5 cm^2^).

### 2.6. Flexural Rigidity

Flexural rigidity is a measure of the stiffness of leather and is related to the bending length of the specimen due to gravitational force [26]. It is usually attributed to the rigidity of collagen fibers in leather [27]. Flexural rigidity was determined according to the Indian standard IS 6490 test method [28]. The samples of dimensions 2.5 × 25 cm^2^ were cut in parallel directions to the backbone of the leather, considering the size of the leather. The rectangular pieces were shorter in length than the specimen length specified in the standard, as it has been observed earlier that the deviation in the length of the samples up to 100 mm does not influence the flexural rigidity. The length of the slacker part of each sample (L) was measured with each side up, first at one end and then at the other, using the constant angle method. The mean value of L was measured for flexural rigidity (G) calculation [27], as is shown below in Equation (1).

(1) $$G=W\times {\left(L\right)}^{3}$$

where:

W = weight per unit area of leather mN/mm

Weight of the leather = mass (g)*gravity in m/s^2^

L = Bending length in mm

G = Flexural rigidity in mN/mm

As shown in Table 3, L1 and L3, L2 and L4 have very similar thickness measurement results and took their result as an average minimum and maximum levels. As calculated, L1 and L2 have lower related rigidity, and L3 and L4 have related higher results, which means:

L1 has a lower thickness, lower rigidity, chrome-tanned.

L2 has a higher thickness, lower rigidity, vegetable tanned, and chrome re-tanned leather.

L3 has lower thickness higher rigidity; this leather is chrome tanned and vegetable re-tanned leather.

L4 has a higher thickness, higher rigidity, and is vegetable-tanned.

Vegetable tanning material gives more thickness, fullness, roundness, etc., whereas chrome gives empty and soft leather.

### 2.7. Drapability

Leather drapability, according to research, is a morphological property that occurs when leather is hung down for gravity [15]. It is one of the essential indicators when measuring close-fitting clothing [29]. The classification of leather as a garment, glove, upper, and so on has a strong influence on its drapability.

There are two ways to examine leather drapes: using your senses or using testers (commonly used). A circular sample with a specific area is placed on a sample clamping plate, with their centers overlapping, causing the drape sample to droop along with the circular plate due to gravity [13].

The drape sample is projected onto a white sheet, and light is used to create the draped figure of the sample, which may be obtained by a shaded area on the ring paper that represents leather drapability. The relevant indexes such as the DC can be obtained by weighing the ring paper and calculating the cut-shaded area. DC is commonly used to assess the drapability of leather and can be calculated in percentage as DC= [(W_2_/W_1_) * 100 percent], which is the projected area ratio to the original area; where W_1_ = total weight of the ring paper (original area) and W_2_ = weight of the shaded area of the ring paper (projected area).

The DC increases as the stiffness of the leather increases and vice versa [30]; thus, the drapability of leather by looking at the drape wave number and amplitude can be estimated. Three samples, each with a diameter of 30 cm and no crease on the surface, are prepared. Each sample should have two sides labeled “a” and “b”, respectively. Three samples, each with a diameter of 30 cm and no crease on the surface, are prepared.

## 3. Results and Discussion

The DC and drapability of the material have an inverse relationship. As the DC becomes large, the drapability of the leather will decrease. As a standard, the garment leather drape should become between the accepted ranges 30–80, where DC below 30 is very limp leather and better to use for very small goods. The DC above 80 is very stiff leather, better to use for upper and safety gloves. The DC largely depends upon the flexural rigidity and thickness of the leather. The DC of sheep garment leather in different levels of the two factors is shown above. As the ANOVA indicates, all factors and their interactions are significant. The DC property between 47.75 and 69.9 largely depends on the thickness of the material. The thickness influences are significantly greater than the flexural rigidity value influences ranging from 94 to 124 sheep garment kinds of leather.

### 3.1. Drape Coefficient vs. Thickness

The thickness of an object is defined as the three descriptive measurements: height, width, and length. The thicknesses of the leathers were measured using a digital thickness tester. The design expert software analysis based on the given data shows thickness on leather drapability. Hence, as a thickness becomes higher, it leads to a higher DC. However, higher thickness in the garment, due to its resulting bulkiness, is often not preferred. The goal of this research was to minimize the thickness to optimize variables.

### 3.2. Drapability vs. Flexural Rigidity

Flexural rigidity is a measure of leather stiffness and is related to the bending length of the specimen. It is usually attributed to the rigidity of the collagen fibers in the leather. The flexural rigidity of garment sheep garment leathers was measured in parallel to the backbone direction. It is seen that there is a significant difference in the flexural rigidity values measured in the two levels ranging from 94 to 124 on DC results between 47 and 69. The DC and flexural rigidity have a positive or direct relationship. These values are significant. As the values of flexural rigidity become high, the DC will be higher, and the drapability will be lower. The plot of DC versus flexural rigidity (calculated from bending length values) reveals that there is a significant change.

### 3.3. Analysis of the Outcome

All key components and the interaction effect have a substantial impact on drapability, according to the analysis of variance (ANOVA), as discussed in Table 4. The 402.60 Model F-values indicate that the model is significant. An F-value of this magnitude has a 0.01% chance of occurring due to noise. While the F-values of rigidity, thickness, and interaction of both factors are significant, the F-values of rigidity, thickness, and interaction of both factors are not. Model terms with P-values less than 0.0500 are significant. Model reduction may improve the model if there are many nominal model terms (not including those required to support hierarchy).

The final model’s projected R^2^ of 0.9852 (98%) agrees reasonably well with the adjusted R^2^ of 0.9910 (99%); that is, the difference is less than 0.2 (20%), implying that the final models can forecast with unpredicted changes of less than 2%, where enough precision measures the signal-to-noise ratio, as it is shown in Table 5.

### 3.4. Model Adequacy Checking

**Figure 1:**
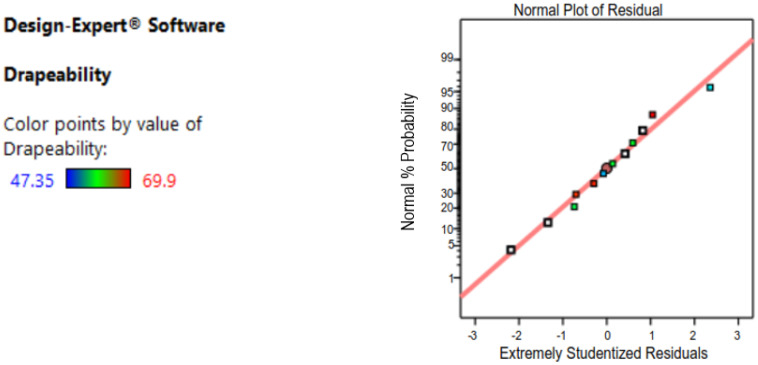
Data analysis for the normal plot of residuals.

**Figure 2:**
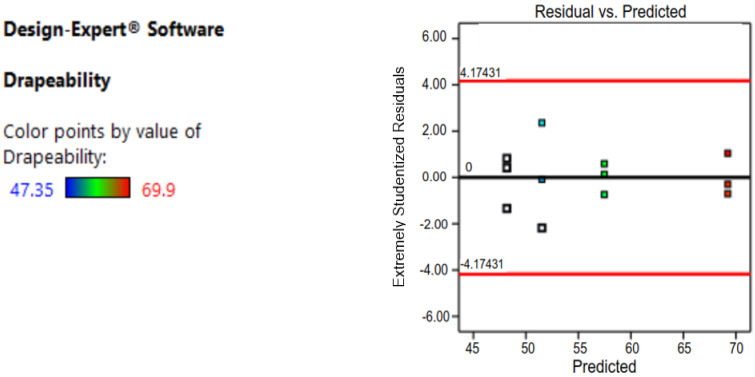
Residual vs. predicted analysis.

Before moving on to the next step, the residuals of the final models must be verified to see if they meet three criteria: first, they must be normally distributed; second, their variance must be constant; and third, they must be independent of the components. The normal probability plot of the residuals, the plot of residuals vs. fitted values, and the plot of residuals versus predicted values of the final models of the response variables, drapability, are shown in Figure 1 and Figure 2. Table 6 shows all the analysis reports.

### 3.5. The Two Factors’ Interaction

**Figure 3:**
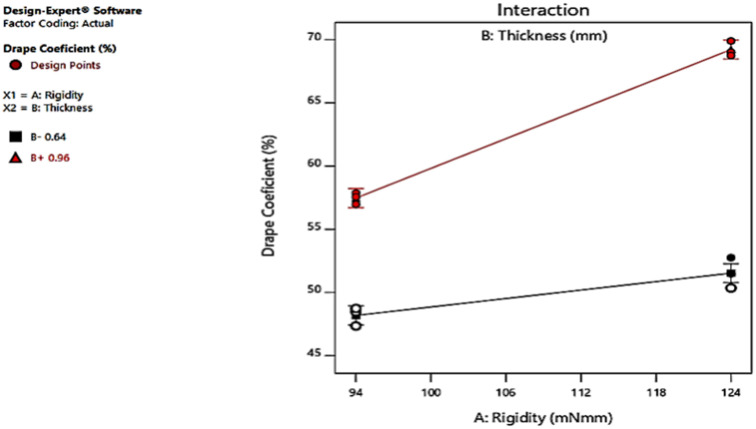
Interaction graph of the two independent variables.

**Figure 4:**
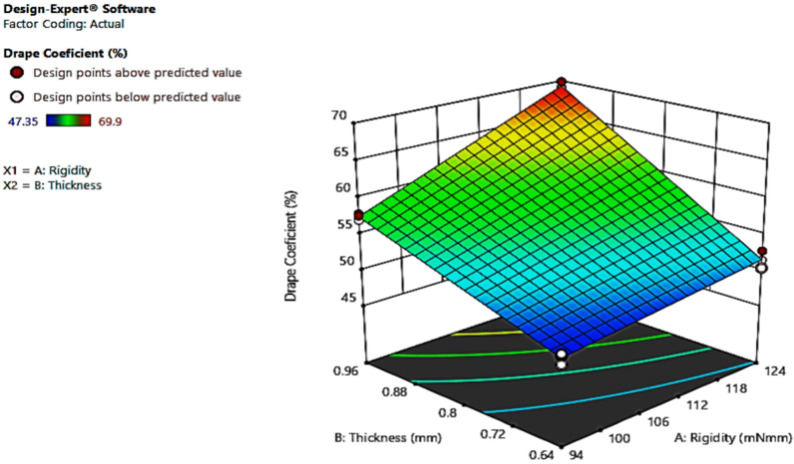
The three-dimensional interaction graph.

The interaction graph implies both factors have an interception at some point as the lines are not parallel, as Figure 3 shows. The graph shows the interaction; the more the thickness becomes, the more the DC. Therefore, the DC and thickness of the leather have a positive or direct relationship. The couture figure shown in Figure 4 represents whether individual factors or their interaction affect the response. Figure 4 represents how both factors’ interaction influences the drapability. This can be identified by whether the couture is curved (has an effect) or a straight line (does not have an effect).

Furthermore, the interaction of both factors is important, as an ANOVA value indicates. The level of one factor affects the other factor. In addition, the two factors’ interaction can be expressed by a three-dimensional graph, as Figure 4 shows.

### 3.6. Model of Regression

The link between the response and the factors can be described in Equation (2) using the final models (in terms of coded factors).

(2) $$Y=B_{0}+B_{1}X_{1}+\;B_{2}X_{2}+B_{12}X_{1}X_{2}=\;56.6\;+\;3.77X_{1}\;+\;6.75X_{2}\;+\;2.1X_{1}X_{2}$$

Here, Y is the response (drapability), B_1_ is factor A (thickness), B_2_ is factor B (rigidity), B_1_B_2_ is the interaction of AB (both factors). Equation (2), in terms of coded factors, can be used to make predictions about the response for given levels of each factor. By default, the high levels of the factors are coded as +1, and the low levels are coded as −1. We obtained the regression model for drapability as A = +3.77, B = +6.75, AB =+2.10 with Intercept = +56.60. The coded equation helps identify the relative impact of the factors by comparing the factor coefficients. This equation can be useful to predict the drapeability level of sheep leather for garments with the minimum standard level of thickness and rigidity within the experimental range of thickness (0.64–0.96 mm) and rigidity (94–124 mN/mm).

### 3.7. Response Optimization

**Figure 5:**
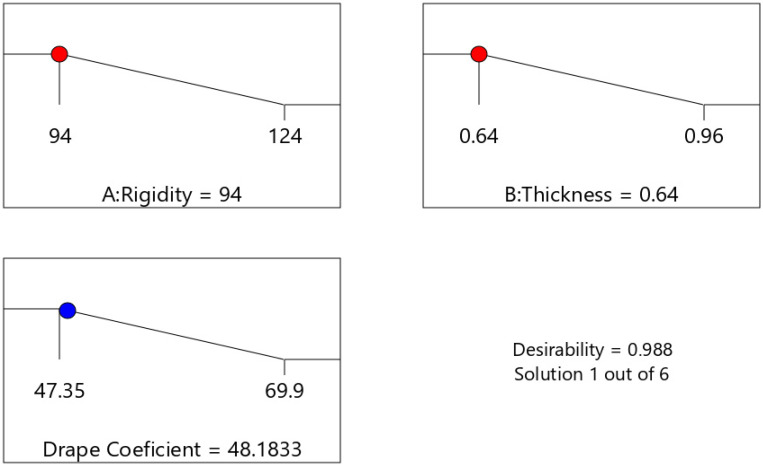
Variables optimization with lower and maximum bounds.

According to the response optimizer in Design-Expert software, the ideal factor setting is 0.64 mm stiffness and 94 mN/mm thickness. Therefore, the required drapability values in this study are set at 48.1833 DC, with lower and maximum bounds of 47.35 DC and 69.9 DC, respectively, as Figure 5 shows.

According to the findings of this study, the relationship between the parameters thickness and rigidity of a sample, and its resistance to drape deformation, is significantly intricate. The specimens sample leathers used in this research have a much smaller thickness and hardness than ideally upper leather (footwear leather) materials, resulting in a bigger drape when the sample is deformed in testing. As leather is not a homogeneous material, tested specimens show that the different portions of the samples have varying thicknesses and rigidities. This observation is highlighted in measuring different parts of the sample. Furthermore, when the four samples are examined at different levels, they reveal a substantial difference. Therefore, when the approximate pure drape model is applied to leather, significant deviations from the theoretical model are inevitable.

## 4. Conclusions

Previous studies studied thickness and rigidity, but they did not study its influence on the drape property of garment leather. This research explains the relationship between sample thickness, rigidity, and drape property and then extends the research by examining the influence of factors that could create a divergence from the ideal drape behavior. The 2^k^ full factorial design is used in this study to determine the appropriate thickness and hardness of garment leather. The results reveal that setting the thickness and rigidity to 0.64 mm and 94 mN/mm, respectively, is the best factor setting for the optimum level of DC of 48.1833%. The DC values can be regulated to be within the required range using this optimization. So, it is possible to conclude that leathers with 0.64 mm thickness and 94 mN/mm rigidity level show an excellent drape that hangs straight down in many little creases and folds. It clings to a bodily part or any other object when placed over it, revealing the form of whatever is beneath it. This study can be used as a benchmark for future studies and a standard reference for clothing makers. The publication has specified the reputability of assessed drapability to reproduce the experiment for other researchers. This study also has practical implications, such as assisting practitioners in comprehending numerous elements and selecting suitable material for garment firms and customers.

## Figures and Tables

**Table 1:** Variables and their levels.

Variables	Standard	Actual Measured Values
Thickness (mm)	0.6–1.0	0.64–0.96
Rigidity/Stiffness (mN/mm)	90–125	94–124
Drape Coefficient (%)	30–80	48–64

**Table 2:** Measurements of leather thickness in mm.

Samples	1	2	3	4	5	6	7	8	9	10	11	12	13	14	15	Average
L1	0.75	0.62	0.58	0.61	0.89	0.63	0.54	0.55	0.56	0.57	0.67	0.58	0.65	0.69	0.65	0.635
L2	0.96	0.93	0.99	1.07	0.95	1.31	0.96	1.05	0.99	0.85	0.82	0.93	0.88	0.90	0.87	0.962
L3	0.71	0.68	0.59	0.60	0.80	0.65	0.55	0.54	0.61	0.58	0.65	0.58	0.65	0.59	0.77	0.6366
L4	0.92	0.97	0.99	1.0	0.93	1.09	0.95	1.15	0.95	0.92	0.93	0.88	0.94	0.91	0.851	0.9601

**Table 3:** Flexural rigidity of sample leathers.

Sample Leathers	Flexural Rigidity (G) in mN/mm
L1	93.96
L2	94.013
L3	124.419
L4	124.55

**Table 4:** ANOVA for the factorial model of choice.

Source	Sum of Squares	Degree of Freedom	Square of Mean	F-Value	*p*-Value	Remarks
Model	770.14	3	256.71	402.60	<0.0001	Significant
A-Rigidity (mN/mm)	170.93	1	170.93	268.07	<0.0001	
B-Thickness (mm)	546.08	1	546.08	856.41	<0.0001	
AB	53.13	1	53.13	83.32	<0.0001	
Pure Error	5.10	8	0.6376			
Cor Total	775.24	11				

**Table 5:** Fit statistics for the model.

**Std. Dev.**	0.7985	R²	0.9934
**Mean**	56.60	Adjusted R²	0.9910
**C.V. %**	1.41	Predicted R²	0.9852
		Adequate Precision	45.6374

**Table 6:** Report of the design analysis.

Run Order	Thickness (mm)	Rigidity (mN/mm)	Actual Value of Drapability	Value Predicted of Drapability	Residual	Residuals That Have Been Externally Studentized	Standard Order
1	0.96	3.4	69.90	69.22	0.6767	1.044	12
2	0.96	1.3	56.97	57.47	−0.4967	−0.740	8
3	0.96	1.3	57.87	57.47	0.4033	0.593	7
4	0.96	3.4	69.02	69.22	−0.2033	−0.294	10
5	0.96	3.4	68.75	69.22	−0.4733	−0.703	11
6	0.64	1.3	48.73	48.18	0.5467	0.821	2
7	0.96	1.3	57.56	57.47	0.0933	0.134	9
8	0.64	1.3	47.35	48.18	−0.8333	−1.340	3
9	0.64	3.4	50.35	51.52	−1.17	−2.182	5
10	0.64	1.3	48.47	48.18	0.2867	0.416	1
11	0.64	3.4	52.75	51.52	1.23	2.357	6
12	0.64	3.4	51.47	51.52	−0.0533	−0.077	4

## Data Availability

Data can be provided by corresponding author on request.
